# Poor early graft function impairs long-term outcome in living donor kidney transplantation

**DOI:** 10.1007/s00345-012-0835-z

**Published:** 2012-02-14

**Authors:** J. Hellegering, J. Visser, H. J. Kloke, F. C. H. D’Ancona, A. J. Hoitsma, J. A. van der Vliet, M. C. Warlé

**Affiliations:** 1 Department of Surgery, Division of Vascular and Transplant Surgery, Radboud University Nijmegen Medical Center, Geert Grooteplein-Zuid 10, 6525 GA Nijmegen, The Netherlands; 2 Department of Nephrology, Radboud University Nijmegen Medical Center, Nijmegen, The Netherlands; 3 Department of Urology, Radboud University Nijmegen Medical Center, Nijmegen, The Netherlands

**Keywords:** Acute rejection, Delayed graft function (DGF), Graft survival, Living donor kidney transplantation (LDKT), Poor early graft function (pEGF), Slow graft function (SGF)

## Abstract

**Background:**

Poor early graft function (EGF) after living donor kidney transplantation (LDKT) has been found to decrease rejection-free graft survival rates. However, its influence on long-term graft survival remains inconclusive.

**Methods:**

Data were collected on 472 adult LDKTs performed between July 1996 and February 2010. Poor EGF was defined as the occurrence of delayed or slow graft function. Slow function was defined as serum creatinine above 3.0 mg/dL at postoperative day 5 without dialysis.

**Results:**

The incidence of slow and delayed graft function was 9.3 and 4.4%, respectively. Recipient overweight, pretransplant dialysis and warm ischemia were identified as risk factors for the occurrence of poor EGF. The rejection-free survival was worse for poor EGF as compared to immediate graft function with an adjusted hazard ratio (HR) of 6.189 (95% CI 4.075–9.399; *p* < 0.001). Long-term graft survival was impaired in the poor EGF group with an adjusted HR of 4.206 (95% CI 1.839–9.621; *p* = 0.001).

**Conclusions:**

Poor EGF occurs in 13.7% of living donor kidney allograft recipients. Both, rejection-free and long-term graft survivals are significantly lower in patients with poor EGF as compared to patients with immediate graft function. These results underline the clinical relevance of poor EGF as phenomenon after LDKT.

## Introduction

Excellent organ quality and ideal transplant conditions contribute to immediate graft function (IGF) in a vast majority of living donor kidney transplantations (LDKT). However, poor early graft function (EGF) still occurs after LDKT, although less frequently than after deceased donor kidney transplantation (DDKT) [1]. Poor EGF includes both delayed graft function (DGF) and slow graft function (SGF). The latter recipients do not have the immediate serum creatinine decrease, but have sufficient EGF to avoid dialysis within the first postoperative week. In previous studies, SGF was defined as a serum creatinine greater than 3 mg/dL on postoperative day 5. The incidence of SGF was found to be 9.5–10.7% after LDKT [2–4]. In another study, the definition of SGF was based on the glomerular filtration rate at postoperative day 14, and SGF occurred in 22.9% [5]. Furthermore, a recent study showed that early graft dysfunction after LDKT may also be defined by the occurrence of delayed posttransplant diuresis [6].

There is clear evidence in DDKT that DGF [7–9] and SGF [10] induce higher immunological activity and impaired renal allograft survival. Also in LDKT, clear evidence exist that both patients with DGF and SGF have higher rates of acute rejection during the first postoperative year as compared to those with IGF [2–5]. However, existing literature is not conclusive whether or not poor EGF impacts long-term allograft survival after LDKT [2–5]. Two retrospective cohort studies did not find a significant correlation between the occurrence of poor EGF and long-term graft survival [2, 5], whereas two other studies did [3, 4]. Therefore, we performed a retrospective cohort study to determine the impact of poor EGF on long-term graft survival.

## Methods

### Patients

Donor and recipient characteristics, clinical data, graft and patient survival status were retrieved from the hospital transplantation database. Laboratory data were collected retrospectively from the hospital electronic patient file. Between July 1996 and February 2010, 520 patients underwent a primary LDKT procedure. In total, 48 of 520 cases were excluded in this study. Criteria for exclusion were: age below 18 years and/or prior kidney transplantation.

### Kidney transplantation procedure

Kidneys were procured by standard open technique using a flank incision until October 1999. From 2001 until 2004, donors were randomized for either a muscle splitting mini incision open or a laparoscopic nephrectomy [11, 12]. Thereafter, laparoscopic nephrectomy was the technique of first choice. Briefly, 4 trocars are introduced using a pneumoperitoneum pressure of 12 mmHg. The renal artery and vein were divided, and the kidney was extracted through a pfannenstiel incision. Kidneys were implanted in the recipient’s iliac fossa through an extraperitoneal approach with vascular anastomosis to the iliac vessels. Extravesicular ureteroneocystostomy was performed, usually with a splint. First warm ischemia time (WIT) was defined as the period between clamping of the renal artery and start of cold perfusion. Second WIT is the time between ending of cold storage and recirculation in the recipient. Cold ischemia time (CIT) is the time between the start of cold perfusion and the beginning of the vascular anastomosis.

### Immunosuppressive protocol

All patients received intravenous methyl-prednisolone administered in the operating room. A vast majority of patients were treated with standard, triple immunosuppressive therapy including a calcineurin inhibitor (CNI), mycophenolate mofetil (MMF) and prednisone. Monoclonal antibody induction (anti-CD25 or ATG) was given to 94 patients in the setting of intervening studies (20%).

### Outcome measures

DGF was defined as the need for dialysis during the first postoperative week. SGF was defined as a serum creatinine above 3.0 mg/dL without the need for dialysis during the first week. All rejection episodes were biopsy-proven. In case of clinical or laboratory evidence of graft dysfunction, doppler ultrasound (duplex) was performed. Biopsy was done immediately in the absence of vascular complications. During episodes of DGF, biopsies were performed at weekly intervals. Failure of the renal allograft was defined as return to another form of renal replacement therapy (dialysis or re-transplantation). Our primary outcome was death-censored renal allograft survival comparing the group with poor EGF (DGF and SGF) to the IGF group.

### Statistical analysis

Continuous variables were given as mean ± standard deviation and were compared using analysis of variance (ANOVA). Categorical variables were given as absolute number of patients and percentages and were compared using Chi-square tests. Patient and graft survival analyses were performed using the Kaplan–Meier method, compared with log-rank tests and adjusted for potential confounders using Cox proportional hazard regression. All available variables were evaluated for potential confounding and included in the multivariate models if a statistically significant effect was demonstrated after entering the Cox proportional hazard model as a single covariate. We used a logistic regression model to calculate odds ratio of risk factors for pEGF, DGF and SGF by multivariate analysis. *p* values <0.05 were considered significant. PASW statistics version 18.0 was used for all analyses (SPSS Inc., Chicago, IL, USA).

## Results

### Incidence of poor EGF

Of all recipients included in this study, 407 showed IGF (86.2%), while 65 experienced poor EGF (13.7%) including 44 (9.3%) patients with SGF and 21 (4.4%) with DGF. In Table 1, baseline donor and recipient characteristics and clinical parameters are presented. Recipients who experienced poor EGF had a significant higher BMI as compared to those with IGF (*p* = 0.011). Also subjects in the poor EGF group had longer WITs, both WIT1 (*p* = 0.043) and WIT2 (*p* = 0.001).

**Table 1:** Recipient and donor characteristics and early graft function

Variables	Whole group	IGF (%)	Poor EGF		*p* value (poor EGF vs. IGF)
			SGF (%)	DGF (%)	
*Number of subjects*	472	407 (86.2)	44 (9.3)	21 (4.4)	
Recipient age (year)	44.8	44.8	44.3	45.1	0.889
Recipient gender male	293 (62.1)	249 (61.2)	33 (75)	11 (52.4)	0.338
Recipient BMI (kg/m^2^)	24.0	23.9	25.1	25.0	0.011
Donor age (year)	50.1	49.9	50.5	52.9	0.351
Donor gender male	212 (44.9)	178 (43.7)	23 (52.3)	11 (52.4)	0.209
Donor BMI (kg/m^2^)	25.5	25.6	25.3	25.2	0.482
Pretransplant dialysis	335 (71)	281 (69)	38 (86.4)	16 (76.2)	0.026
HLA mismatches	3.2	3.2	3.4	3.7	0.172
>1 renal artery	14	11 (2.7)	1 (2.3)	2 (9.5)	0.423
WIT1 (min)	3.4	3.3	3.7	4.5	0.043
WIT2 (min)	29.8	29.0	32.2	39.6	0.001
Total CIT (min)	132	132	126	152	0.754
Right kidney	114 (24.2)	96 (23.6)	12 (27.3)	6 (28.6)	0.533
Laparoscopic	277 (58.7)	234 (57.5)	25 (56.8)	18 (85.7)	0.222
Serum creat (μmol/L)					
2 weeks	157	130	292	442	<0.001
1 month	137	128	194	211	<0.001
1 year	128	124	159	143	<0.001
*Disease etiology*					
Diabetes	22	18	3	1	0.508
Polycystic kidney	49	45	2	2	0.366
Glomerulosclerosis	8	8	0	0	0.604
Glomerulonephritis	41	34	7	0	0.458
Pyelonephritis	24	19	3	2	0.218
*Initial maintenance immunosuppression*					
MoAb induction	94	76	10	8	0.096
Tacrolimus	346	301	28	17	0.451
Mycophenolate	433	372	40	0	0.633
Sirolimus	13	12	1	0	1.000

### Rejection-free survival

**Fig. 1:**
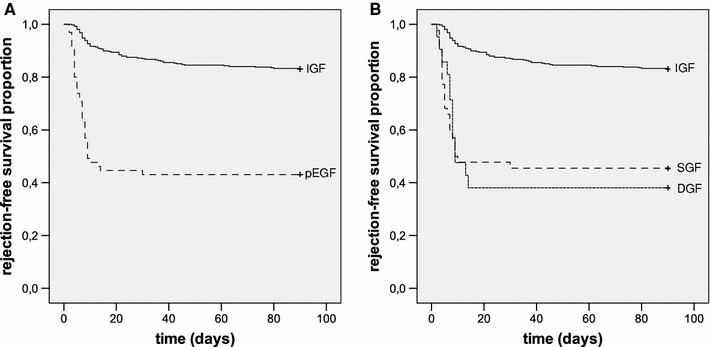
**a** Rejection-free survival during the first 90 days after LDKT in patients with IGF versus poor EGF (*p* < 0.001). **b** rejection-free survival in patients with IGF versus SGF (*p* < 0.001) and IGF versus DGF (*p* < 0.001)

Rejection-free survival during the first ninety postoperative days was worse for poor EGF as compared to the IGF group (Fig. 1a; log-rank, *p* < 0.001). This difference in rejection-free survival persisted after adjusting for potential confounders in a Cox proportional hazard model, with an adjusted hazard ratio (HR) of 6.189 (95% CI 4.075–9.399; *p* < 0.001). The rejection-free survival of the SGF and DGF groups both differed significantly from the IGF group (Fig. 1b; *p* < 0.001).

### Death-censored graft survival

**Fig. 2:**
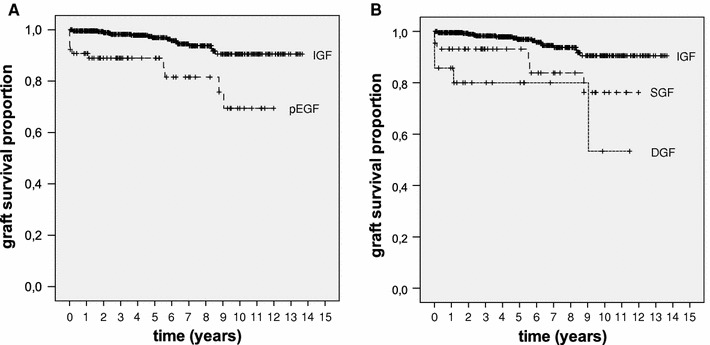
**a** Death-censored graft survival after LDKT in patients with IGF versus poor EGF (*p* < 0.001). **b** death-censored graft survival in patients with IGF versus SGF (*p* < 0.001) and IGF versus DGF (*p* < 0.001)

There are no significant differences in patient survival between the poor EGF and IGF groups, nor between the DGF, SGF and IGF groups. In Fig. 2 the death-censored renal allograft survival is presented. Survival was worse in the poor EGF group (Fig. 2a), and the difference was highly significant with an adjusted HR of 4.206 (95% CI 1.839–9.621; *p* = 0.001). Figure 2b shows death-censored graft survival for the SGF and DGF groups as compared to the IGF group (log-rank *p* < 0.001). When SGF was compared to IGF, there was a significant difference with an adjusted HR of 3.619 (95% CI 1.403–9.337; *p* = 0.008). For the difference between DGF and IGF, also a significance was found with an adjusted HR of 6.340 (95% CI 1.832–21.938; *p* = 0.004).

### Risk factors of poor EGF

A univariate and multivariate logistic regression analysis was performed to reveal risk factors for poor EGF. Only those variables that reached statistical significance in univariate analysis were included in the multivariate logistic regression model. Recipient BMI, pretransplant dialysis, WITs 1 and 2 were significant predictors of poor EGF.

### Laparoscopic procurement and graft outcome

**Fig. 3:**
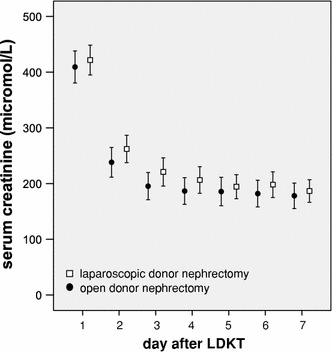
Mean serum creatinine levels in recipients of living donor kidney allografts procured by laparoscopic (*n* = 277) versus open (*n* = 195) techniques

Figure 3 shows mean serum creatinine levels during the first week after LDKT in recipients of allografts procured by laparoscopic versus open techniques. Although serum creatinine levels appeared to be slightly higher in recipients of laparoscopically procured kidneys, differences were not significant. Furthermore, recipients of laparoscopically procured kidney allografts did not show significantly higher incidences of poor EGF (Table 1). Cox regression analysis showed that the occurrence of poor EGF impaired long-term graft survival in recipients of kidneys procured by both laparoscopic and open techniques, with HRs of 4.642 (95% CI 1.478–14.58; *p* = 0.008) and 3.795 (95% CI 1.313–10.97; *p* = 0.014), respectively.

## Discussion

This study shows that poor EGF following LDKT has a large impact on long-term graft survival. Recipients with poor EGF have a fourfold risk of graft failure as compared to those with IGF. Recipients with SGF display almost four times the risk of graft failure as compared to those with immediate function; those with DGF have a sixfold risk of graft failure. Although the phenomenon of SGF appears less dramatic as compared to DGF, both have a substantial deleterious impact on graft survival as compared to the IGF group. These results are in concordance with recent studies by Nogueira and Tyson et al. [3, 4], both describing a retrospective cohort of kidney transplant recipients, all receiving laparoscopically procured living donor kidneys. The percentage of laparoscopically procured kidneys in two other studies by Brennan and Lee et al. [2, 5] was 22 and 82%, respectively; both studies did not find a significant association between poor EGF and graft survival. In our cohort, 59% received a laparoscopically procured kidney, and the occurrence of poor EGF affected long-term survival in recipients of kidneys after open donor nephrectomy also. This finding is in line with a recent report on LDKT after open nephrectomy in which a correlation was found between delayed posttransplant diuresis and impaired long-term graft outcome [6]. Altogether, we believe that our data show that the deleterious effect of poor EGF on long-term graft survival applies for recipients of living donor kidneys after laparoscopic and also after open donor nephrectomy.

Multivariate analysis of covariates revealed four significant risk factors for poor EGF, including recipient BMI, pretransplant dialysis and warm ischemia. Recipient BMI was also identified as a predictor of poor EGF by Nogueira et al. [3]. These findings are in line with recent reports showing that higher recipient BMI is associated with DGF [13, 14]. An explanation for the association between recipient BMI and poor EGF may be that the implantation of especially right kidneys (usually with shorter renal veins) into obese recipients is technically more challenging, resulting in prolonged anastomosis times that may contribute to the occurrence of poor EGF. Pretransplant dialysis has been identified as a significant risk factor for poor EGF. A possible explanation for this finding may be that in patients on dialysis creatinine values prior to transplantation are higher as compared to preemptive transplanted patients. Although creatinine levels converge between those groups after transplantation, patients who were on pretransplant dialysis have significantly higher serum creatinine levels at day 5 (data not shown). Prolonged warm ischemia was also revealed as a significant predictor for poor EGF by Brennan and Nogueira et al. [2, 3]. Results from this study provides additional evidence that a prolonged warm ischemia is an important determinant of poor EGF. Interestingly, our data confirm previous findings [2, 5] indicating that the type of donor procurement (laparoscopic or open nephrectomy) does not affect the incidence of poor EGF. Furthermore, we observed slightly higher serum creatinine values after LDKT of laparoscopically procured kidneys (Fig. 3), but these differences were not statistically significant. Although a minimal deleterious influence of the pneumoperitoneum on EGF could not be ruled out, our data suggest that its impact is confined.

Limitations of this study are mainly related to its retrospective design. Since all consecutive kidney transplant recipients entered the transplantation database and there was almost no loss to follow-up, the risk of selection bias is low. Since patients experiencing poor EGF were more likely to receive a biopsy, some degree of observational bias could not be ruled out. In other words, the observed higher detection rate of (subclinical) rejection as compared to patients with IGF may be explained by the activated (innate) immune response in patients with early graft dysfunction, but also by a higher likelihood to receive a renal biopsy. Further prospective studies are required to clarify this issue. Although we controlled for many potential confounders in the statistical analyses, some degree of confounding cannot be ruled out. For example, cardiovascular comorbidity may influence EGF, but also long-term graft and patient survival. Despite these limitations, we conclude that our findings underline the need to develop strategies to reduce the rate of poor EGF after LDKT. These strategies may include (remote) ischemic preconditioning to reduce the deleterious effects of renal ischemia–reperfusion injury [15] and further shortening of WITs.

## Abbreviations

CIT: cold ischemia time
WIT: warm ischemia time
DGF: delayed graft function
pEGF: poor early graft function
SGF: slow graft function
DDKT: deceased donor kidney transplantation
LDKT: living donor kidney transplantation
HLA: human leukocyte antigen
